# Supplementary material for: Complementary shifts in photoreceptor spectral tuning unlock the full adaptive potential of ultraviolet vision in birds
Source: eLife. 2016 Jul 12;5:e15675. doi: 10.7554/eLife.15675 (PMC4947394; doi:10.7554/eLife.15675)
Supplement: Supplementary file 1. — The tuning of the SWS1 opsin is inferred from the amino acid at position 90 of the second transmembrane helix (Ödeen and Håstad, 2013; 2009). The amino acid sequence was either derived from previously published studies or was determined by sequencing of genomic DNA in the current study as indicated. DOI: http://dx.doi.org/10.7554/eLife.15675.020 [file elife-15675-supp1.docx]

**Supplementary File 1.** The species included in our phylogenetic comparison of retina apocarotenoid composition. The tuning of the SWS1 opisin is inferred from the amino acid substitutions at AA90 of transmembrane region 2. The amino acid sequence was determined form previously published studies or sequencing of genomic DNA in the current study as indicated.

| Catalog No. | **Species** | **Order** | **SWS1 amino acid sequence** | **Visual system** | **Dihydrogalloxanthin :galloxanthin ratio** | **SWS1 sequence source** | **Specimen Database URL** |
| --- | --- | --- | --- | --- | --- | --- | --- |
| N/A | *Struthio camelus* | Struthioniformes | FISCIFSVFTV | VS | 0.44 | Odeen and Hastad 2003 |  |
| N/A | *Coturnix japonica* | Galliformes | FVSCVLSVFVV | VS | 0 | Odeen and Hastad 2003 |  |
| N/A | *Gallus gallus* | Galliformes | FVSCVLSVFVV | VS | 0.29 | Odeen and Hastad 2003 |  |
| MSB:Bird:33163 | *Penelope montagnii* | Galliformes | GFVSCSFSVFIV | VS | 0.479 | Current study | http://arctos.database.museum/guid/MSB:Bird:33163 |
| N/A | *Anas platyrhynchos* | Anseriformes | FVSCIFSVFIV | VS | 0 | Odeen and Hastad 2003 |  |
| MSB:Bird:33191 | *Campephilus haematogaster* | Piciformes | LSCIFSVFTV | VS | 0.674 | Current study | http://arctos.database.museum/guid/MSB:Bird:33191 |
| MSB:Bird:33118 | *Colaptes rupicola* | Piciformes | GFLSCIFSVFTV | VS | 0.368 | Current study | http://arctos.database.museum/guid/MSB:Bird:33118 |
| N/A | *Coccyzus americanus* | Cuculiformes | GFVSCIFSVFTV | VS | 0.139 | Current study |  |
| MSB:Bird:33502 | *Metriopelia melanoptera* | Columbiformes | GFISCIFSVFTV | VS | 0.395 | Current study | http://arctos.database.museum/guid/MSB:Bird:33502 |
| N/A | *Zenaida macroura* | Columbiformes | GFISCIFSVFTV | VS | 0.387 | Current study |  |
| MSB:Bird:33268 | *Amazilia chionogaster* | Trochiliformes | LCCIFSVFTV | VS | 0.386 | Current study | http://arctos.database.museum/guid/MSB:Bird:33268 |
| MSB:Bird:33312 | *Patagona gigas* | Trochiliformes | FISCIFSVFTV | VS | 0.576 | Current study | http://arctos.database.museum/guid/MSB:Bird:33312 |
| MSB:Bird:28532 | *Agelaius phoeniceus* | Passeriformes | LXCCVFCIFTV | UV | 0.791 | Current study | http://arctos.database.museum/guid/MSB:Bird:28532 |
| MSB:Bird:33465 | *Anairetes reguloides* | Passeriformes | MCCIFSVFTV | VS | 0.664 | Current study | http://arctos.database.museum/guid/MSB:Bird:33465 |
| MSB:Bird:33171 | *Cacicus chrysonotus* | Passeriformes | LMCCVFCIFTV | UV | 0.745 | Current study | http://arctos.database.museum/guid/MSB:Bird:33171 |
| MSB:Bird:28516 | *Calcarius ornatus* | Passeriformes | LMCCVFCIFTV | UV | 0.75 | Current study | http://arctos.database.museum/guid/MSB:Bird:28516 |
| N/A | *Carpodacus mexicanus* | Passeriformes | LMCCVFCIFTV | UV | 0.769 | Current study |  |
| MSB:Bird:33242 | *Catamblyrhynchus diadema* | Passeriformes | CCVFCIFTV | UV | 0.845 | Current study | http://arctos.database.museum/guid/MSB:Bird:33242 |
| MSB:Bird:33093 | *Cnemarchus erythropygius* | Passeriformes | FMCCIFSVFTV | VS | 0.419 | Current study | http://arctos.database.museum/guid/MSB:Bird:33093 |
| MSB:Bird:33308 | *Conirostrum cinereum* | Passeriformes | CCVFCIFTV | UV | 0.895 | Current study | http://arctos.database.museum/guid/MSB:Bird:33308 |
| MSB:Bird:33257 | *Cyanolyca viridicyanus* | Passeriformes | GFLCCIFSVFTV | VS | 0.345 | Current study | http://arctos.database.museum/guid/MSB:Bird:33257 |
| MSB:Bird:33103 | *Diglossa brunneiventris* | Passeriformes | GLMCCVFCIFTV | UV | 0.707 | Current study | http://arctos.database.museum/guid/MSB:Bird:33103 |
| MSB:Bird:33265 | *Elaenia albiceps* | Passeriformes | LCCIFSVFTV | VS | 0.708 | Current study | http://arctos.database.museum/guid/MSB:Bird:33265 |
| MSB:Bird:33245 | *Entomodestes leucotis* | Passeriformes | LCCVFCIFTV | UV | 0.707 | Current study | http://arctos.database.museum/guid/MSB:Bird:33245 |
| N/A | *Erythrura gouldiae* | Passeriformes | MSP | UV | 0.911 | Hart et. al. 2000a |  |
| MSB:Bird:33217 | *Grallaria rufula* | Passeriformes | FLCCLFSVFTV | VS | 0.955 | Current study | http://arctos.database.museum/guid/MSB:Bird:33217 |
| MSB:Bird:33168 | *Lepidocolaptes lacrymiger* | Passeriformes | FLCCIFSVFTV | VS | 0.555 | Current study | http://arctos.database.museum/guid/MSB:Bird:33168 |
| MSB:Bird:33239 | *Margarornis squamiger* | Passeriformes | FMCCIFSVFTV | VS | 0.793 | Current study | http://arctos.database.museum/guid/MSB:Bird:33239 |
| MSB:Bird:33204 | *Mionectes striaticollis* | Passeriformes | FFCCIFSVFTV | VS | 0.79 | Current study | <http://arctos.database.museum/guid/MSB:Bird:33204> |
| MSB:Bird:33221 | *Myioborus melanocephalus* | Passeriformes | GLCCCVFCIFTV | UV | 0.756 | Current study | http://arctos.database.museum/guid/MSB:Bird:33221 |
| MSB:Bird:33225 | *Nephelomyias ochraceiventris* | Passeriformes | FMCCIFSVFTV | VS | 0.749 | Current study | http://arctos.database.museum/guid/MSB:Bird:33225 |
| MSB:Bird:33226 | *Ochthoeca pulchella* | Passeriformes | MCCIFSVFTV | VS | 0.796 | Current study | http://arctos.database.museum/guid/MSB:Bird:33226 |
| MSB:Bird:28548 | *Passer domesticus* | Passeriformes | LMCCVFCIFTV | UV | 0.769 | Current study | <http://arctos.database.museum/guid/MSB:Bird:28548> |
| MSB:Bird:33281 | *Phrygilus fruticeti* | Passeriformes | GLMCCVFCIFTV | UV | 0.845 | Current study | http://arctos.database.museum/guid/MSB:Bird:33281 |
| MSB:Bird:33387 | *Phrygilus punensis* | Passeriformes | LMCCVFCIFTV | UV | 0.659 | Current study | http://arctos.database.museum/guid/MSB:Bird:33387 |
| MSB:Bird:33104 | *Poospiza caesar* | Passeriformes | LCCVFCIFTV | UV | 0.77 | Current study | http://arctos.database.museum/guid/MSB:Bird:33104 |
| N/A | *Sturnus vulgaris* | Passeriformes | LMCCIFCIFTV | UV | 0.732 | Odeen and Hastad 2003 |  |
| MSB:Bird:33173 | *Synallaxis azarae* | Passeriformes | GFLCCIFSVFTV | VS | 0.931 | Current study | http://arctos.database.museum/guid/MSB:Bird:33173 |
| N/A | *Taeniopygia guttata* | Passeriformes | GLMCCVFCIFTV | UV | 0.875 | Summer 2012 |  |
| MSB:Bird:33205 | *Thraupis cyanocephala* | Passeriformes | GLMCCVFCIFTV | UV | 0.888 | Current study | http://arctos.database.museum/guid/MSB:Bird:33205 |
| MSB:Bird:33181 | *Pheugopedius eisenmanni* | Passeriformes | LMCCIFCIFTV | UV | 0.798 | Current study | http://arctos.database.museum/guid/MSB:Bird:33181 |
| MSB:Bird:33363 | *Upucerthia validirostris* | Passeriformes | LCCIFSVFTVF | VS | 0.716 | Current study | http://arctos.database.museum/guid/MSB:Bird:33363 |
| MSB:Bird:33110 | *Zonotrichia capensis* | Passeriformes | LCCVFCIFTV | UV | 0.866 | Current study | http://arctos.database.museum/guid/MSB:Bird:33110 |
| MSB:Bird:33121 | *Bolborhynchus orbygnesius* | Psittacidae | FLACIFCIFTV | UV | 1 | Current study | http://arctos.database.museum/guid/MSB:Bird:33121 |
| MSB:Bird:33192 | *Hapalopsittaca melanotis* | Psittacidae | MCCVFCIFTV | UV | 0.914 | Current study | http://arctos.database.museum/guid/MSB:Bird:33192 |
