# Supplementary material for: Complementary shifts in photoreceptor spectral tuning unlock the full adaptive potential of ultraviolet vision in birds
Source: eLife. 2016 Jul 12;5:e15675. doi: 10.7554/eLife.15675 (PMC4947394; doi:10.7554/eLife.15675)
Supplement: Supplementary file 2. — DOI: http://dx.doi.org/10.7554/eLife.15675.021 [file elife-15675-supp2.docx]

**Supplementary file 2.** The species and visual system parameters used to model avian color discrimination.

|  |  |  | Visual pigment λ_cut_ (nm) | | | | Ocular Media λ_T0.5_ (nm) | Oil droplet λ_cut_ | | |  |
| --- | --- | --- | --- | --- | --- | --- | --- | --- | --- | --- | --- |
| Species | Species | Visual System | SWS1 | SWS2 | MWS | LWS |  | C-type | Y-type | R-type | Reference |
| Blackbird | *Turdus merula* | UVS | 373 | 454 | 504 | 557 | 343 | 414 | 515 | 570 | (^1,2,3^Hart et al. 2000a) |
| Blue tit | *Parus caeruleus* | UVS | 372 | 449 | 502 | 563 | 317 | 413 | 508 | 573 | ^(1,2,3^Hart et al. 2000a) |
| Budgerigar | *Melopsittacus undulatus* | UVS | 371 | 440 | 499 | 566 | 320 | 411 | 497 | 568 | (^1^Bowmaker et al. 1997; ^2^Knott, et al., 2012; ^3^Lind et al., 2014) |
| Canary | *Serinus canaria* | UVS | 363 | 440 | 501 | 567 | 321 | 414 | 506 | 578 | (^1,2^Das et al. 1999; ^3^Lind et al., 2014*) |
| Cut-throat finch | *Amadina fasciata* | UVS | 370 | 447 | 500 | 563 | 318 | 423 | 516 | 575 | (^1,2,3^Hart et al. 2000b) |
| Gouldian finch | *Erythrura gouldiae* | UVS | 370 | 440 | 500 | 562 | 317 | 422 | 513 | 572 | (^1,2,3^Hart et al. 2000b) |
| White-headed munia | *Lonchura maja* | UVS | 373 | 446 | 500 | 562 | 318 | 422 | 510 | 567 | (^1,2,3^Hart et al. 2000b) |
| Red-billed leothrix | *Leiothrix lutea* | UVS | 355 | 454 | 499 | 568 | 321 | 392 | 506 | 566 | (^1,2^Maier 1993; ^3^Lind et al., 2014*) |
| Plum-headed finch | *Neochmia modesta* | UVS | 373 | 442 | 500 | 562 | 316 | 415 | 514 | 568 | (^1,2,3^Hart et al. 2000b) |
| Starling | *Sturnus vulgaris* | UVS | 362 | 449 | 504 | 563 | 338 | 399 | 515 | 573 | (^1,2,3^Hart et al. 1998) |
| Zebra finch | *Taeniopygia guttata* | UVS | 359 | 427 | 505 | 566 | 321 | 414 | 510 | 571 | (^1,2^Bowmaker et al. 1997; ^3^Lind et al., 2014) |
| Bowerbird | *Ptilonorhynchus violaceus* | VS | 410 | 450 | 509 | 563 | 343 | 430 | 518 | 570 | (^1,2,3^Coyle et al. 2012) |
| Chicken | *Gallus gallus* | VS | 418 | 453 | 507 | 571 | 351 | 443 | 505 | 561 | (^1,2^Bowmaker et al. 1997; ^3^Lind and Kelber, 2009) |
| Mallard duck | *Anas platyrhynchos* | VS | 415 | 452 | 506 | 567 | 371 | 445 | 506 | 561 | (^1,2,3^Jane and Bowmaker 1988) |
| Peafowl | *Pavo cristatus* | VS | 424 | 458 | 505 | 567 | 365 | 449 | 511 | 569 | (^1,2,3^Hart 2002) |
| Pigeon | *Columba livia* | VS | 404 | 452 | 506 | 566 | 337 | 448 | 514 | 586 | (^1,2^Bowmaker et al. 1997;  ^3^Lind and Kelber, 2009) |
| Japanese quail | *Coturnix japonica* | VS | 418 | 450 | 505 | 567 | 352 | 446 | 511 | 566 | (^1,2^Bowmaker et al. 1993; ^3^Lind et al., 2014*) |
| Wedge-tailed shearwater | *Puffinus pacificus* | VS | 406 | 450 | 503 | 566 | 335 | 445 | 506 | 562 | (^1,2,3^Hart 2004) |

^1^Reference for visual pigment absorbance, ^2^reference for oil droplet absorbance, ^3^reference for ocular media transmittance, *ocular media transmittance is approximated using an average transmittance spectra for UVS or VS species, see Lind et al. for further details.

**Supplementary file 2.** **References:**

Bowmaker, J. K,. Heath, L. A., Wilkie, S. E. & Hunt, D. M. 1997 Visual pigments and oil droplets from six classes of photoreceptor in the retinas of birds. *Vision Res*. **37**, 2183–2194.

Bowmaker, J. K., Kovach J. K., Whitmore A. V. & Loew, E. R. 1993￼Visual pigments and oil droplets in genetically manipulated and carotenoid deprived quail: A mircospectrophotometric study. *Vision Res*. **33**, 571-578.

Das, D., Wilkie, S. E., Hunt, D. M., Bowmaker, J. K. 1999 Visual pigments and oil droplets in the retina of a passerine bird, the canary *Serinus canaria*: microspectrophotometry and opsin sequences. *Vision Res*. **39**, 2801-2815.

Hart, N. S. 2002 Vision in the peafowl (Aves: *Pavo cristatus*). *J. Exp. Biol*. **205**, 3925 – 3935.

Hart, N. S. 2004 Microspectrophotometry of visual pigments and oil droplets in a marine bird, the wedge-tailed shearwater *Puffinus pacificus*: topographic variations in photoreceptor spectral characteristics. *J. Exp. Biol*. **207**, 1229 – 1240. (doi:10.1242/jeb.00857)

Hart, N. S., Partridge, J. C., Cuthill, I. C. 1998 Visual pigments, oil droplets and cone photoreceptor distribution in the European starling (*Sturnus vulgaris*). *J. Exp. Biol*. **201**, 1433 – 1446.

Hart, N. S., Partridge, J. C., Cuthill, I. C. & Bennett, A. T. D. 2000a Visual pigments, oil droplets, ocular media and cone photoreceptor distribution in two species of passerine bird: the blue tit (*Parus caeruleus* L.) and the blackbird (*Turdus merula* L.). *J Comp Physiol A* **186,** 375–387

Hart, N. S., Partridge, J. C., Bennett, A. T. D. & Cuthill, I. C. 2000b Visual pigments, cone oil droplets and ocular media in four species of estrildid finch. *J Comp Physiol A* **186,** 681–694.

Jane, S. D. & Bowmaker, J. K. 1988 Tetrachromatic colour vision in the duck (*Anas platyrhynchos* L.): microspectrophotometry of visual pigments and oil droplets. *J. Comp. Physiol. A* **162**, 225 – 235. (doi:10.1007/BF00606087)

Knott, B., Bowmaker, J. K., Berg, M. L. & Bennett, A. T. D. 2012 Absorbance of retinal oil droplets of the budgerigar: sex, spatial and plumage morph-related variation. *J Comp Physiol A* **198**, 43-51. (doi: 10.1007/s00359-011-0684-z)

Lind O. & Kelber, A. 2009 Avian colour vision: effects of variation in receptor sensitivity and noise data on model predictions as compared to behavioural results. *Vision Res*. **49**, 1939–1947. (doi:10.1016/ j.visres.2009.05.003)

Lind, O., Mitkus, M., Olsson, P. & Kelber, A. 2014 Ultraviolet vision in birds: the importance of transparent eye media. *Proc. Biol. Sci.* **281**, 20132209. (doi:10.1098/rspb.2013.2209)

Maier, E. J. & Bowmaker, J. K. 1993 Colour vision in the passeriform bird, *Leiothrix lutea*: correlation of visual pigment absorbance

and oil droplet transmission with spectral sensitivity. *J Comp Physiol A.* **172**, 295-301.
