# Supplementary material for: Complementary shifts in photoreceptor spectral tuning unlock the full adaptive potential of ultraviolet vision in birds
Source: eLife. 2016 Jul 12;5:e15675. doi: 10.7554/eLife.15675 (PMC4947394; doi:10.7554/eLife.15675)
Supplement: Supplementary file 3. — (a) PCR primers used to clone in situ hybridization templates. (b) Primers used for qPCR quantification of apocarotenoid-metabolizing enzyme transcript expression in developing chicken retinas. (c) PCR primers used to clone full-length transcripts of apocarotenoid-metabolizing enzymes for cloning into the pTre expression vector. DOI: http://dx.doi.org/10.7554/eLife.15675.022 [file elife-15675-supp3.docx]

**Supplementary File 3a.** PCR primers used to clone *in situ* templates

| Primer | Sequence* | Template length (bp) |
| --- | --- | --- |
| BCO2_In_Situ_917F | gctaccgctcgagATAAACGCACTGGGAAGGTG | 600 |
| BCO2_In_Situ_1516R | gctaccgctcgagCAACGGACAAGATGACTCCA |  |
| RDH12_In_Situ_236F | gctaccggaattcTGATTGTTGCCTGCAGAGAC | 586 |
| RDH12_In_Situ_821R | gctaccggaattcCCTTCCCATGGAGTCTTCAA |  |
| RetSat_In_Situ_826F | gctaccggaattcATCCTGGTGCAGCACTACCT | 999 |
| RetSat_In_Situ_1824R | gctaccggaattcGTTCTTCTTCTTGCCGTTGG |  |

*The coding sequence is given in upper case and the appended restriction site in lower case.

**Supplementary File 3b.** Primers used for qPCR quantification of apocarotenoid metabolizing enzyme transcript expression in developing chicken retinas.

| Primer | Sequence |
| --- | --- |
| Gg_BCO2_qPCR_F | ACGGCAGAAGACAGTGGAGT |
| Gg_BCO2_qPCR_R | CTCTGCATCCAAGACAAGCA |
| Gg_RDH12_qPCR_F | GTTATGCCCTTACTCCAAGACA |
| Gg_RDH12_qPCR_R | CTTCAGGCGCTCCAGTAATAA |
| Gg_RetSat_qPCR_F | CGCTTACGAGGACGTAAAGAA |
| Gg_RetSat_qPCR_R | ATGTAGTGCTGGTTGGTCAG |
| Gg_GAPDH_qPCR_F | GAGGGTAGTGAAGGCTGCTG |
| Gg_GAPDH_qPCR_R | TGGCTGTCACCATTGAAGTC |

**Supplementary File 3c.** PCR primers used to clone full-length transcripts of apocarotenoid metabolizing enzymes for cloning into the pTre expression vector.

| Primer | Sequence* | Restriction site |
| --- | --- | --- |
| Gg_BCO2_F | ggtaccgacatATGCAATTCGTCCCAGGTCT | *Nde*I |
| Gg_BCO2_R | ggtaccgatgtacaGTTCTTCTTCTTGCCGTTGGAG | *Bsr*GI |
| Gg_RDH12_F | tctacagagctagcATGGAGCCGGCGGCG | *Nhe*I |
| Gg_RDH12_R | ggtaccgagaattcGCAGGAGGCTGTACTGCTCA | *Eco*RI |
| Tg_RetSat_F | ggtaccgacatATGTGGCTGCAGGCGCT | *Nde*I |
| Tg_RetSat_R | ggtaccgatgtacaGTCTCCCTTCTTGGAGCTGG | *Bsr*GI |

*The coding sequence is given in upper case and the appended restriction site in lower case.
